# RNA-Seq Reveals Waterlogging-Triggered Root Plasticity in Mungbean Associated with Ethylene and Jasmonic Acid Signal Integrators for Root Regeneration

**DOI:** 10.3390/plants11070930

**Published:** 2022-03-30

**Authors:** Jaruwan Sreeratree, Pimprapai Butsayawarapat, Tanapon Chaisan, Prakit Somta, Piyada Juntawong

**Affiliations:** 1 Department of Genetics, Faculty of Science, Kasetsart University, Bangkok 10900, Thailand; jaruwan.sre@ku.th (J.S.); pimprapai.bu@ku.th (P.B.); 2 Department of Agronomy, Faculty of Agriculture, Kasetsart University, Bangkok 10900, Thailand; fagrtpc@ku.ac.th; 3 Department of Agronomy, Faculty of Agriculture at Kamphaeng Saen, Kasetsart University, Nakhon Pathom 73140, Thailand; agrpks@ku.ac.th; 4 Center for Advanced Studies in Tropical Natural Resources, National Research University-Kasetsart University, Bangkok 10900, Thailand; 5 Omics Center for Agriculture, Bioresources, Food and Health, Kasetsart University (OmiKU), Bangkok 10900, Thailand

**Keywords:** ethylene, jasmonic acid, root plasticity, waterlogging, RNA-seq

## Abstract

Global climate changes increase the frequency and intensity of heavy precipitation events, which result in flooding or soil waterlogging. One way to overcome these low-oxygen stresses is via modifying the plant root system to improve internal aeration. Here, we used a comparative RNA-seq based transcriptomic approach to elucidate the molecular mechanisms of waterlogging-triggered root plasticity in mungbean (*Vigna radiata*), a major grain legume cultivated in Asia. Two mungbean varieties with contrasting waterlogging tolerance due to the plasticity of the root system architecture were subjected to short-term and long-term waterlogging. Then, RNA-seq was performed. Genes highly expressed in both genotypes under short-term waterlogging are related to glycolysis and fermentation. Under long-term waterlogging, the expression of these genes was less induced in the tolerant variety, suggesting it had effectively adapted to waterlogging via enhancing root plasticity. Remarkably, under short-term waterlogging, the expression of several transcription factors that serve as integrators for ethylene and jasmonic acid signals controlling root stem cell development was highly upregulated only in the tolerant variety. Sequentially, root development-related genes were more expressed in the tolerant variety under long-term waterlogging. Our findings suggest that ethylene and jasmonic acids may contribute to waterlogging-triggered root plasticity by relaying environmental signals to reprogram root regeneration. This research provides the basis for the breeding and genetic engineering of waterlogging-tolerant crops in the future.

## 1. Introduction

Global climate changes increase the frequency of adverse weather, which pertinently challenges worldwide agriculture. One of the most significant problems resulting from global climate changes is flooding, which can be classified as waterlogging (WS; when the height of the water column covers the root zone) or as submergence (when the aerial parts are fully covered [1,2]. WS habitually affects dryland crops because soil can become easily waterlogged due to poor drainage after heavy rainfall or irrigation. WS results in a hypoxic environment in the roots due to the limited gas diffusion [3]. Eventually, plants that experience WS will suffer energy shortage from the inhibition of oxidative phosphorylation. Long-term WS causes stomatal closure, leading to impaired root hydraulic conductivity, reduced photosynthesis, and the inhibition of plants’ nutrient and water uptake [4].

Mungbean (*Vigna radiata*) is an important short-season subtropical grain legume commonly grown in Asia. Mungbean seeds and sprouts are nutritious, providing a source of protein, vitamins, and minerals, while plant parts can be used as fodder and green manure [5]. The mungbean growing area covers 7.3 million hectares, and the global output is around 5.3 million tons [6]. Although mungbean is considered moderately tolerant to heat and drought stress, it is highly sensitive to WS, especially at the early growth stages [7,8]. Since mungbean is typically grown as relay crops in the rice field, excess soil moisture in the rice field could expose mungbean seedlings to WS [7]. Therefore, in the world of increasing food demand, WS tolerance is a key for mungbean breeding programs.

To improve mungbean WS tolerance, it is essential to understand the tolerance mechanism attributed to WS. In general, two main strategies allow plants to cope with waterlogging, namely the escape strategy and the quiescence strategy. In the escape strategy, plants utilize the storage carbohydrate to develop adventitious roots (AR) and form aerenchyma, promoting gas transportation [9,10]. On the contrary, the quiescent strategy relies on energy conservation to minimize growth until the water level subsides [9,11]. A recent study has shown that mungbean could develop AR and lateral root (LR) under prolonged WS, but the ability to form AR was varied among the varieties [7]. Our previous study of WS-tolerant zombi pea (*Vigna vexillata*) demonstrated that the auxin-regulated LR development plays an essential role in mitigating the impact of WS [12]. Similarly, a major quantitative trait locus (QTL), *qWT_Gm_03*, controlling WS-induced root plasticity, was identified in soybean and proposed to be involved in the auxin pathways regulating secondary root development and root plasticity [13]. In flood-tolerant bittersweet *Solanum dulcamara*, ethylene entrapped by WS is a major regulator of flooding-induced AR primordium and stem cell tissue activation [14]. These lines of evidence indicate that the integration of hormone signaling pathways could regulate the WS-triggered root plasticity. Nonetheless, the molecular mechanism controlling WS-triggered root plasticity is not well understood in plants.

In this study, we performed RNA-seq using two mungbean varieties that are different in developing WS-triggered root plasticity. Comparative transcriptome profiling of roots and hypocotyls subjected to short-term and long-term WS was analyzed. We hypothesized that the natural genetic diversity would allow us to find the molecular mechanism controlling WS-triggered root plasticity.

## 2. Results

### 2.1. H150 Waterlogging Tolerance Associates with Modification of Root Architecture

**Figure 1:**
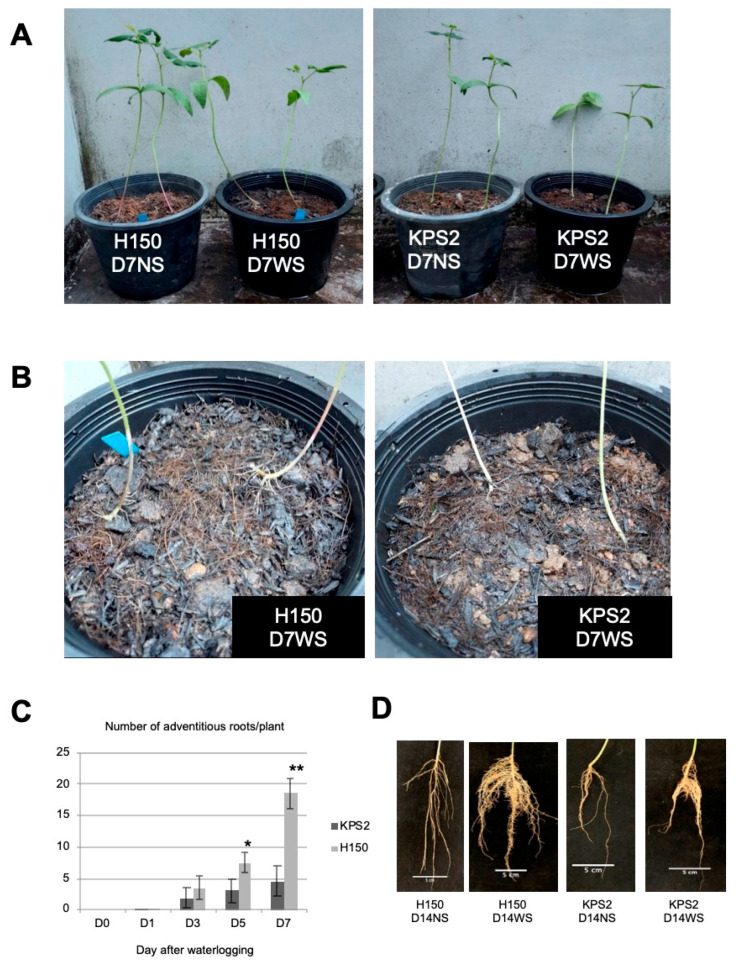
Contrasting phenotypes of H150 and KPS2 varieties under WS. (**A**) Representative 15-day-old seedlings subjected to 7 days (D7) of waterlogging (WS) or kept for 7 days under control (NS). (**B**) Formation of adventitious roots after seven days WS. (**C**) Time-course analysis of adventitious roots induced by WS. *n* = 8 plants; error bars = SE; * *p* < 0.05; ** *p* < 0.01 (*t*-test). (**D**) Whole root phenotype under 14 days (D14) of WS.

Two mungbean varieties, H150, the WS-tolerant landrace variety collected from Thailand where crop plants are subjected to frequent waterlogging, and Kamphaeng Saen 2 (KPS2), the WS-sensitive commercial variety, were chosen based on the contrasting phenotype in response to WS. Initially, a screening experiment was conducted by growing over 100 mungbean varieties in pot soils and WS for 30 days (data not shown). In this study, WS was applied to the 15-day-old seedlings. Under seven-day (D7) WS, H150 maintained better growth than KPS2 based on a visual examination (Figure 1A).

Since root plasticity plays a vital role in the adaptation to WS, we observed that WS significantly induced adventitious root (AR) formation in H150 compared to KPS2 (Figure 1B,C). Time-course analysis of AR formation demonstrated that ARs began to emerge after one day (D1) WS. At day 5 (D5) WS, the average number of ARs of H150 was significantly higher than KPS2 (Figure 1C). At D7 WS, 18.6 ARs were found in H150, while only 4.5 ARs were found in KPS2 (Figure 1C). The continuation of WS for 14 days (D14) revealed that the enhanced AR numbers and lateral roots (LR) development were observed from H150 compared to KSP2 (Figure 1D). Together, these data suggest that root plasticity could be an essential determinant for waterlogging tolerance in mungbean.

### 2.2. Transcriptome Reconfiguration in Mungbean Subjected to Waterlogging

For the transcriptomic study, three biological replicates of two WS time points, D1 (short-term) and D7 (long-term), and a day 1 no stress (NS) time point were included in this study. To perform differential gene expression analysis, reads were mapped to the previously reported mungbean reference genome. The majority of reads (95–97%) of each RNA-seq library could be mapped to the reference (Table S1), suggesting the reliability of our RNA-seq data. The number of reads aligned to each gene was acquired for differential gene expression analysis using edgeR software. Differentially expressed genes (DEGs) from seven comparisons (D1H150/NSH150, D1KPS2/NSKPS2, D7H150/NSH150, D7KPS2/NSKPS2, NSH150/NSKPS2, D1H150/D1KPS2, and D7H150/D7KPS2) were calculated. DEGs with significant changes in gene expression evaluated by the false discovery rate (FDR) < 0.05 and having at least one comparison with a log2 fold change > 1 or <−1 were retained for further analysis (Table S2). Of seven comparisons, four comparisons, namely D1H150/NSH150, D1KPS2/NSKPS2, D7H150/NSH150, and D7KPS2/NSKPS2, were calculated to identify waterlogging responsive DEGs. In contrast, the other three comparisons, namely NSH150/NSKPS2, D1H150/D1KPS2, and D7H150/D7KPS2, were calculated to compare gene expression levels among the two varieties.

**Figure 2:**
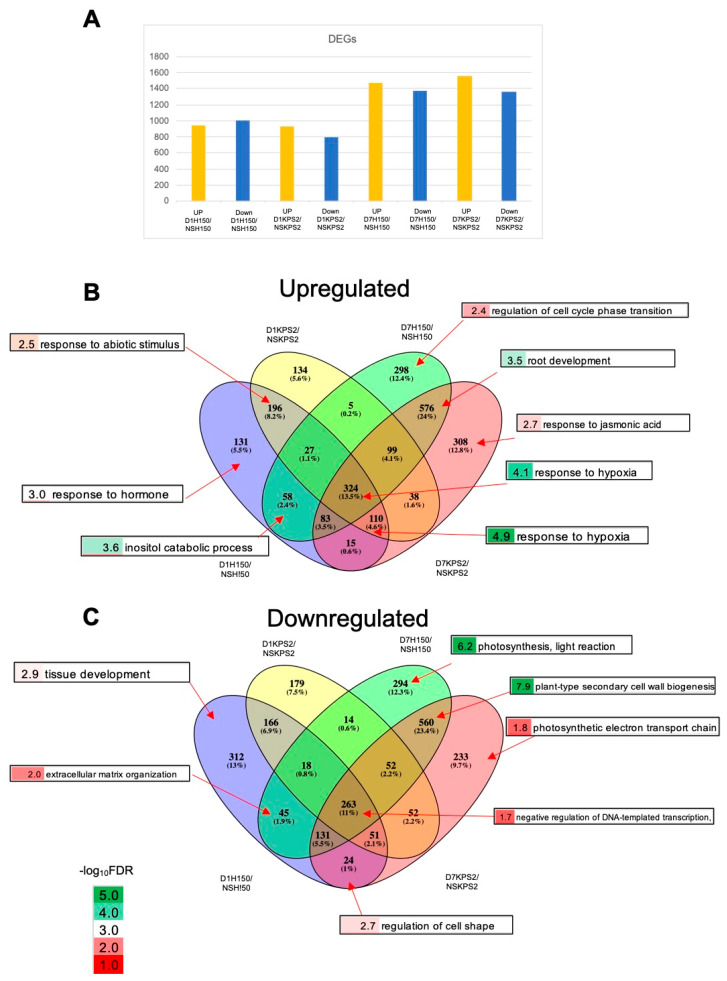
WS altered root transcriptomes of “H150” and “KPS2” varieties. (**A**) The number of upregulated and downregulated differentially expressed genes (DEGs) from roots of “H150” and “KPS2” in response to 1 day (D1) and 7 days (D7) of WS. Venn’s diagram of (**B**) upregulated and (**C**) downregulated DEGs, and the top enrichment biological process GO terms in response to WS. Data used to generate this figure can be found in Tables S2 and S4.

The numbers of waterlogging responsive DEGs are D1H150/NSH150: 1954; D1KPS2/NSKPS2: 1728; D7H150/NSH150:2847; and D7KPS2/NSKPS2: 2919 (Figure 2A). D7WS led to nearly 1.5 times DEGs, indicating that an extended WS period strongly affects the adaptive response.

Venn diagram analysis of up- and downregulated DEGs were generated (Figure 2B,C). Of 2402 upregulated DEGs, 1182 were remarkably upregulated at D7WS, 759 DEGs were upregulated under D1WS and D7WS, and a minor number (461) of DEGs were upregulated at D1WS (Figure 2B). Similarly, in the case of down-regulated DEGs, of 2422, a majority (1087 DEGs) were downregulated at D7WS, 678 DEGs were down-regulated under both D1WS and D7WS, and 657 DEGs were downregulated at D7WS (Figure 2C).

Based on the Venn diagram, gene ontology (GO) enrichment analysis was performed for each category. To annotate the mungbean genes, we identified their Arabidopsis orthologs (Table S3) and obtained associated GO terms. Enriched GO terms (FDR cutoff < 0.05) for each category can be found in Table S4. As expected, for the upregulated DEGs, the top-scored enriched biological process is “response to hypoxia” (Figure 2B; Table S4). Interestingly, this term was commonly found in all four comparisons (“324”). However, “110” DEGs were upregulated in all comparisons, except for D7H150/NSH150 which had also functioned in “response to hypoxia”, indicating that H150 was better adapted to prolonged WS. For the down-regulated DEGs (Figure 2C), the top-scored enriched biological process is “plant-type secondary cell wall biogenesis” (Figure 2C; Table S4).

**Figure 3:**
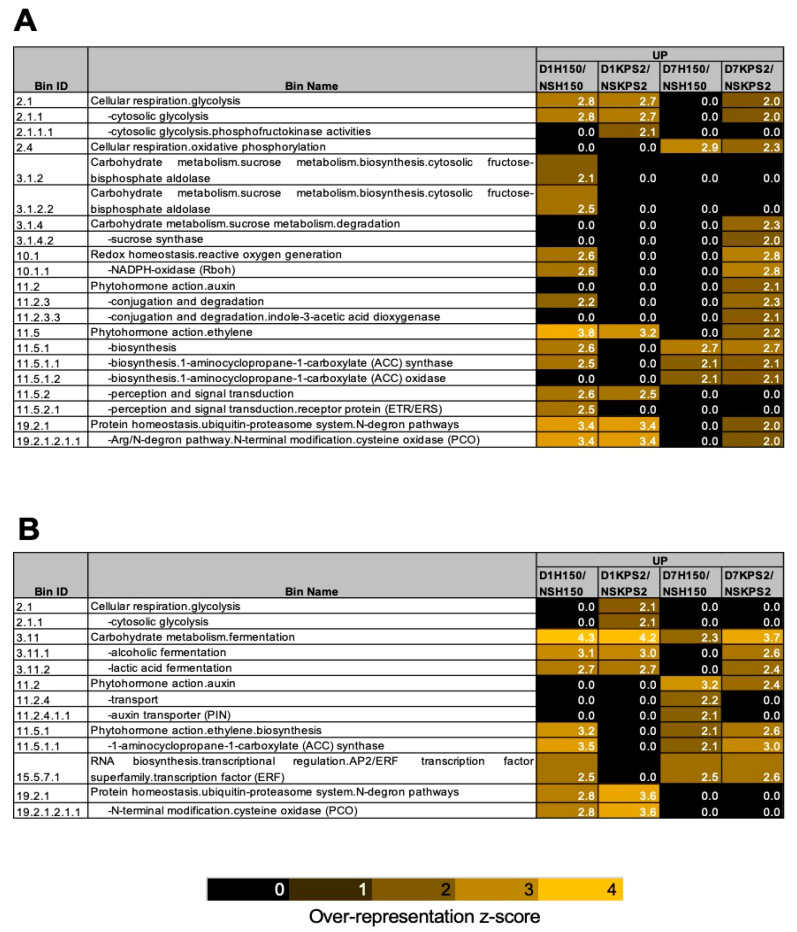
PAGEMAN over-representation analysis of DEGs (for selected functional categories). (**A**) Log2 Fold Change cutoff = 1. (**B**) Log2 Fold Change Cutoff = 2. Data used to generate this figure can be found in Table S5.

To identify the cellular process associated with WS response in mungbean, we further analyzed the transcriptome using a comparative transcriptomic approach by over-representation analysis (ORA) with Fisher’s exact test. Two different log2 fold change cutoff values, a cutoff value of one (weak differential expression) and a cutoff value of two (strong differential expression), were applied (Table S5; Figure 3A,B). Using the cutoff value of 1 (Figure 3A), the ORA analysis demonstrated that glycolysis (Bin 2.1) and the N-degron pathway genes, PCOs (plant cysteine oxidases; Bin 19.2.1.2.1.1), were over-represented in the upregulated DEGs of D1H150/NSH150, D1KPS2/NSKPS2, and D7KPS2/NSKPS2 but not in D7H150/NSH150. Oxidative phosphorylation (Bin 2.4) was more over-represented in the upregulated DEGs of D7H150/NSH150 than D7KPS2/NSKPS2. Sucrose biosynthesis (Bin 3.1.2) was over-represented in the upregulated DEGs of D1H150/NSH150, while sucrose degradation (Bin 3.1.4) was over-represented only in the upregulated DEGs of D7KPS2/NSKPS2. Reactive oxygen generation (Bin 10.1) and auxin conjugation and degradation (Bin 11.2.3) were over-represented in the upregulated DEGs of D1H150/NSH150 and D7KPS2/NSKPS2. Interestingly, ethylene biosynthesis enzymes, ACC (1-aminocyclopropane-1-carboxylate) synthase (Bin 11.5.1.1) were over-represented in the upregulated DEGs of D1H150/NSH150, D7H150/NSH150, and D7KPS2/NSKPS2 but not in D1KPS2/NSKPS2. When using the cutoff value of 2 (Figure 3B), alcoholic fermentation (Bin 3.11.1) and lactic acid fermentation (Bin 3.11.2) were over-represented in the upregulated DEGs of D1H150/NSH150, D1KPS2/NSKPS2, and D7KPS2/NSKPS2 but not in D7H150/NSH150. Auxin transport (Bin 11.2.4) was specifically over-represented in the upregulated DEGs of D7H150/NSH150. ACC synthase (Bin 11.5.1.1) and ERF transcription factor (Bin 15.5.7.1) were over-represented in the upregulated DEGs of D1H150/NSH150, D7H150/NSH150, and D7KPS2/NSKPS2 but not in D1KPS2/NSKPS2. Glycolysis (Bin 2.1.1) and PCOs (Bin 19.2.1.2.1.1) were more over-represented in the upregulated DEGs of D1KPS2/NSKPS2 than D1H150/NSKPS2. These data confirm that H150 and KPS2 reprogramed their transcriptome in response to WS and could utilize distinct WS adaptive strategies.

### 2.3. Differential Regulation of Gene Associated with Anaerobic Fermentation and Glycolysis

**Figure 4:**
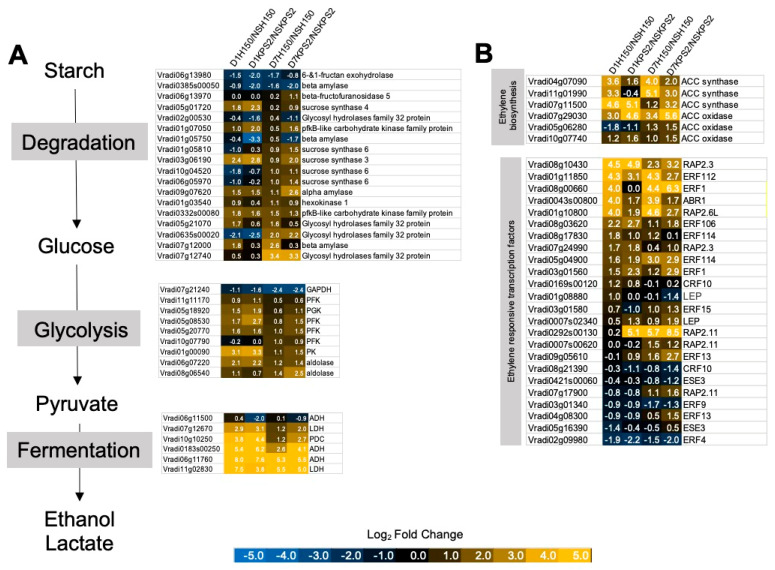
WS resulted in differential expression of transcripts encoding proteins involved in energy and ethylene production. (**A**) major carbohydrate metabolism, glycolysis, fermentation (**B**) ethylene biosynthesis and response. The number indicates log2 fold changes. Data can be found in Table S2.

Since the GO enrichment and ORA results suggested that glycolysis and fermentative gene expression were distinctively regulated upon WS in the two varieties, we sought to examine the expression patterns of these particular DEGs (Figure 4A). We observed the upregulation of many starch degradation and glycolysis genes in both varieties following D1WS. D7WS further enhanced the expression level of the sucrose synthases (*Vradi01g05810*, *Vradi03g06190*, *Vradi05g01720*, *Vradi06g059710*, and *Vradi10g04520*) and maintained the glycolysis gene expression in KPS2. However, their expression level in H150 was lower than that of KPS2. Similarly, in the case of anaerobic fermentative genes, most of them were upregulated in both varieties under D1WS. In contrast, under D7WS, the expression level of ALCOHOL DEHYDROGENASE (*ADH: Vradi06g11500* and *Vradi0183s00250*), LACTIC ACID DEHYDROGENASE (*LDH: Vradi07g12670*), and PYRUVATE DECARBOXYLASE (*PDC*: *Vradi10g10250*) genes was lower than that of KPS2.

### 2.4. Differential Regulation of Gene Associated with Ethylene Production and Response

Ethylene is regarded as one of the essential hormones controlling plant adaptation to WS. Based on RNA-seq results, we found several DEGs encoding for proteins functioning in ethylene synthesis and response (Figure 4B). In general, ACC synthase and ACC oxidase genes were upregulated by WS. However, the expression levels of two ACC synthase genes (*Vradi04g07090* and *Vradi11g01990*) were higher in H150 than KPS2 under both D1WS and D7WS. We calculated the total count per million (CPM) expression values for the three ACC synthases (*Vradi04g07090*, *Vradi11g01990*, and *Vradi07g11500)* (Figure S1). However, under NS, KPS2 demonstrated higher CPM values of ACC synthases than H150 (3.2 vs. 1.6, respectively). WS resulted in higher CPM values of ACC synthases in H150 than KPS2 (D1H150: 24.5; D7H150: 27.1; D1KPS2: 20.6; D7KPS2: 17.2), suggesting that H150 could produce more ethylene under WS.

Ethylene mediates physiological, developmental, and stress responses by activating ethylene response factors (ERFs). Our results demonstrated that many *ERFs* were differentially expressed following WS. Interestingly, three members of ERF subfamily X (*ERFX*; *Vradi01g11850* (*ERF112*), *Vradi0043s00800* (*ABR1*), and *Vradi01g10800* (*RAP2.6L*)) were more highly expressed in H150 than KPS2 under both D1WS and D7WS. The expression of *ERF1* (*Vradi08g00660*) was upregulated following D1WS in H150 but not in KPS2. Additionally, two members of the ERF VII subfamily (*ERFVII*: *Vradi08g10430* and *Vradi07g24990*) were upregulated following D1WS in both varieties. Nonetheless, at D7WS, their expression in H150 was less than in KPS2.

### 2.5. Differential Regulation of Genes Associated with NO Production, Primary Nitrogen Metabolism, and Hypoxia Sensing and Response

**Figure 5:**
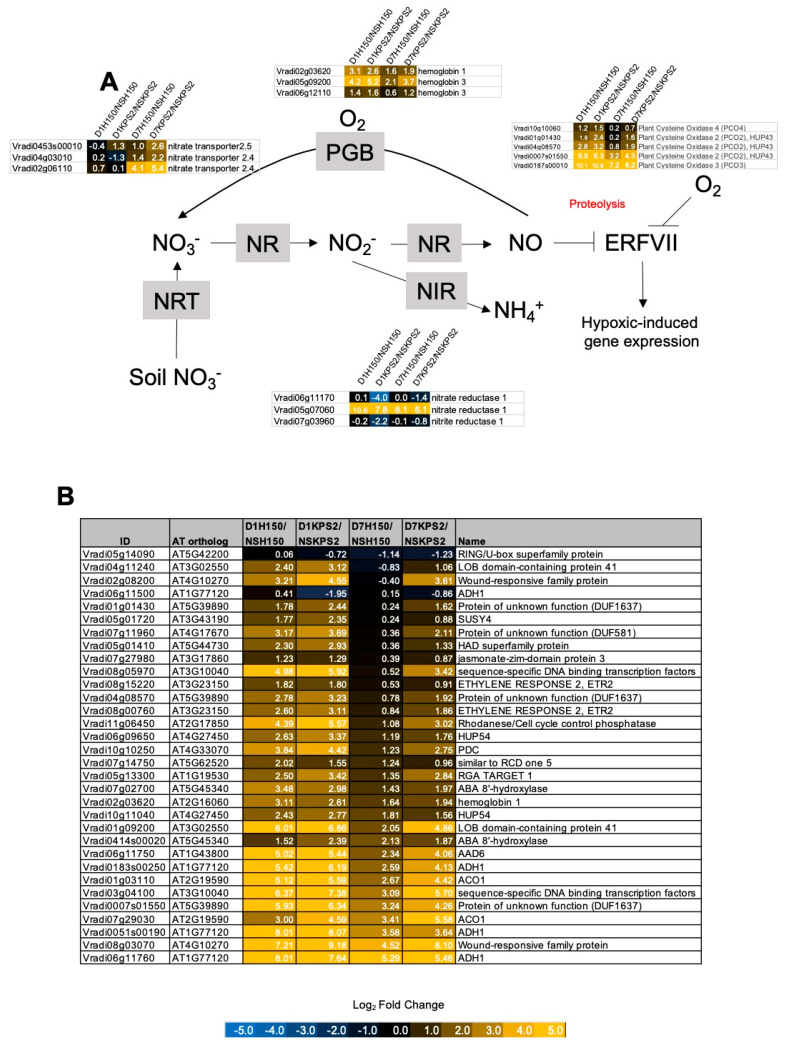
WS resulted in differential expression of NO production, primary nitrogen metabolism, low-oxygen sensing, and core hypoxia response genes. (**A**) NO production, primary nitrogen metabolism, and low-oxygen sensing genes (**B**) Core hypoxia genes. The number indicates log2 fold changes. Data can be found in Table S2.

Since nitrate could improve plant tolerance to low-oxygen stress, we examined the expression of genes associated with nitrogen metabolism and NO production (Figure 5A). The expression of nitrate transporter genes was strongest induced in D7KPS2/NSKPS. The expression of an enzyme responsible for NO production, NITRATE REDUCTASE (*NR*: *Vradi05g07060*), was higher in H150 than KPS2 under D1WS and D7WS. The expression of NO scavenging enzyme, phytoglobins (*PGBs*), and oxygen sensing enzyme, *PCOs*, was induced in both D1H150/NSH150 and D1KPS2/NSKPS2. However, under D7WS, the expression of *PGBs* and *PCOs* was higher in KPS2 than in H150. Both *PGBs* and *PCOs* are primary targets of ERFVIIs controlling plant low-oxygen response. We also observed a strikingly similar pattern when the expression of evolutionarily conserved core hypoxia-responsive genes was examined (Figure 5B). This group is also the primary target of ERFVIIs.

### 2.6. Validation of RNA-Seq Results

**Figure 6:**
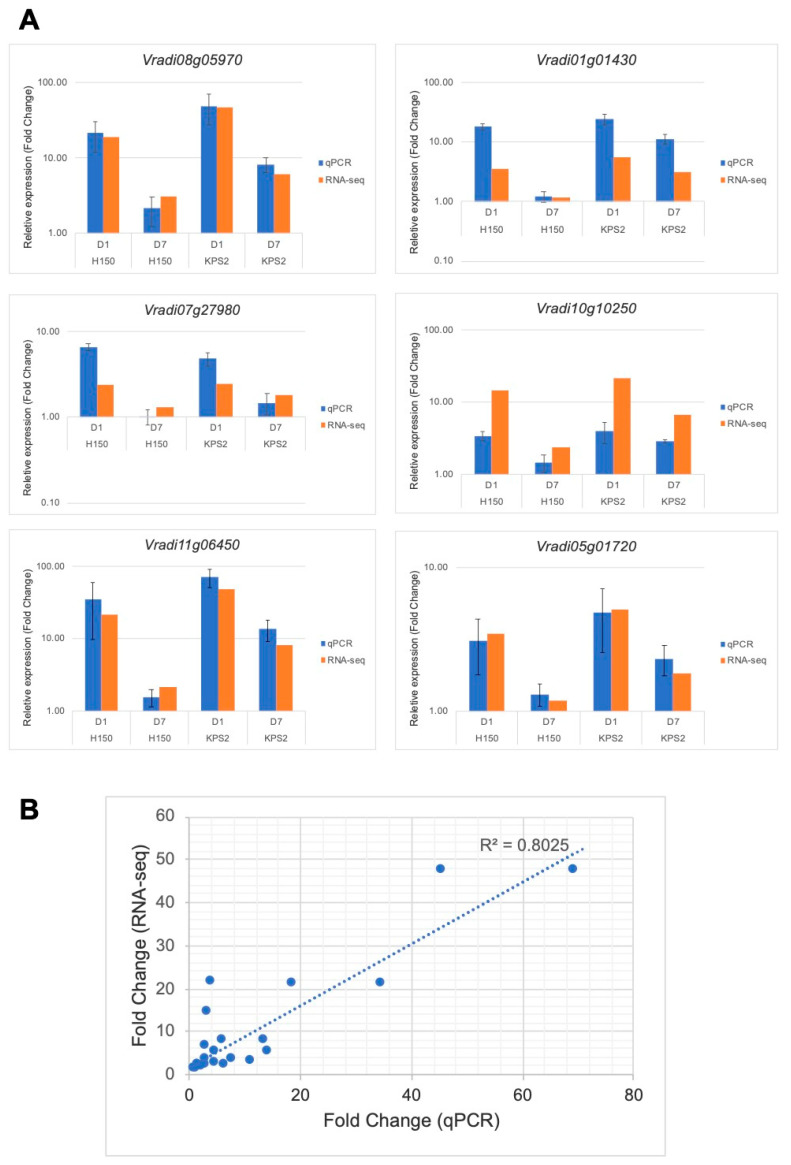
qPCR validation of RNA-seq data. (**A**) Comparison between qPCR and RNA-seq results for six DEGs. (**B**) Correlation between RNA-seq and qPCR data derived from six DEGs. Relative expression was normalized to the abundance of *UBQ10 (Vradi0167s00180*). Data represent mean ± SE (*n* = 3).

To validate the RNA-seq results, quantitative real-time PCR analysis (qPCR) was performed using a set of primers specific for six hypoxia-responsive genes (*Vradi05g01720: SUSY*; *Vradi08g05970: HRA1*; *Vradi07g27980: Jasmonate-zim-domain protein 3*; *Vradi10g10250: PDC1*; *Vradi01g01430: DUF163*; *Vradi11g06450: Rhodanese*). We observed a similar pattern of regulation of both RNA-seq and qPCR results (Figure 6A). Regression analysis of RNA-seq and qPCR results was performed (Figure 6B). We found a positive correlation between these two data sets (r^2^ = 0.80). Overall, the results confirm the reliability of our RNA-seq data.

### 2.7. Cluster Analysis Reveals Early Induction of Stress Responsive Genes and Late Induction of Developmental Genes in the Tolerant Variety

**Figure 7:**
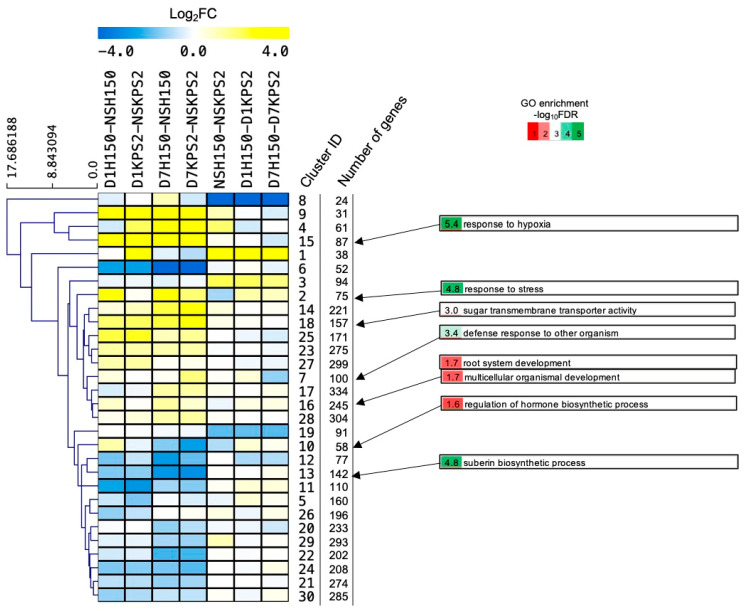
Fuzzy k-mean analysis of DEGs from roots of “H150” and “KPS” subjected to WS. The heat map shows median log_2_ fold change values for 30 clusters of transcripts with similar regulation. Selected enriched GO terms were displayed. Data can be found in Table S6.

Construction of the gene expression regulatory network requires the identification of similarly expressed genes. To identify the molecular mechanism controlling root plasticity under WS, DEGs were subjected to fuzzy k-mean cluster analysis (Figure 7; Table S6). The fuzzy algorithm is considered a soft clustering algorithm since it allows DEGs to belong to more than one cluster. As a result, it can be used to identify genes controlled by more than one factor and genes that may encode a protein with more than one function. All seven DEG comparisons were included in our analysis, and the number of clusters and membership exponent was initially determined by trial and error. Finally, a total of 30 clusters was obtained. Then, GO enrichment analysis was performed to identify the associated functions (Table S7). Some selected clusters are described here. Cluster#2 genes associated with “response to stress” were strongly induced by WS in D1H150/NSH150, D7H150/NSH150, and D7KPS/NSKPS2 but not in D1KPS2/NSKPS2, with a higher abundance in D1H150 and D7H150 than D1KPS2 and D7KPS2, respectively. Cluster#7 genes that demonstrated a contrasting pattern since they were induced by WS only in D1H150/NSH150 and D7KPS/NSKPS2 were associated with “defense response to other organisms”. Cluster#10 genes induced by WS only in D1H150/NSH150 but repressed by WS in D7H150/NSH150 and D7KPS2/NSKPS2 were associated with “regulation of hormone biosynthetic process”. Cluster#13 genes repressed by WS in all samples were associated with “suberin biosynthetic process”. Cluster#15 genes, which were strongly induced by WS in all samples, but whose abundance was higher in D7KPS2 than D7H150, functioned in “response to hypoxia”. Cluster#16 genes have a similar gene expression pattern to Cluster#2 but with a lesser degree of WS induction and a slightly more robust induction at D7WS were related to “root system development” and “multicellular organismal development”. Cluster#18 genes were strongly induced by WS in both H150 and KPS2, and their abundance appeared equal in the two varieties. Cluster#18 genes were associated with “sugar transmembrane transporter activity”.

**Figure 8:**
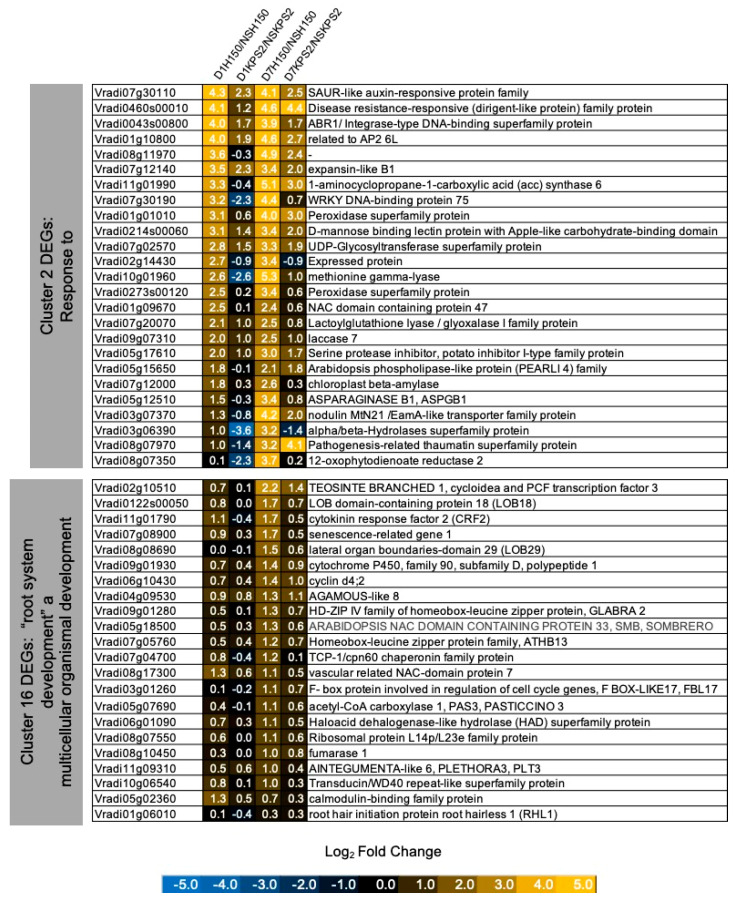
Differential expression of DEGs involves in regulation of stress response and development. Expression pattern of selected DEGs from cluster 2 (transcription regulators) and cluster 16 (multicellular organismal development and Leucine-rich kinases). Data can be found in Table S2.

Based on the pattern of gene expression and the GO term enrichment results, we placed our attention on Cluster#2 genes associated with “response to stress” and Cluster#16 genes related to “root system development” and “multicellular organismal development” (Figure 8). Interestingly, jasmonic acid (JA) and ethylene-responsive transcription factors that can activate root stem cells, *ABR1* and *RAP2.6L,* were found in Cluster#2. In addition, a key enzyme for ethylene biosynthesis, ACC synthase (*Vradi11g01990*), was also in Cluster#2. Two direct targets of auxin response factor (ARF) 7 and 19, LATERAL ORGAN BOUNDARIES DOMAIN 18 and 29 (*LBD18*: *Vradi0122s00050; LBD29: Vradi08g08690*), were found in Cluster#16. Several auxin-related genes with roles in the AR and LR formation, PLETHORA 3 (*PLT3*: *Vradi11g09310*), CYTOKININ RESPONSE FACTOR 2 (*CRF2: Vradi11g01790*), and SOMBRERO *(SMB: Vradi05g18500),* were also found in Cluster#16.

## 3. Discussion

Root plasticity plays an essential role in adapting to WS because the root is the organ directly subjected to low-oxygen stress underground. This study analyzed RNA-seq to compare the differential gene expression profiles of two mungbean varieties with contrasting WS tolerance at D1WS and D7WS. Our results show that WS tolerance could be attributed to modifying root system architecture upon experiencing WS (Figure 1). The improved AR and LR formation correlates with the WS-tolerant trait in several plant species [12,15,16,17,18]. WS adaptation resulted in the reprogramming of the mungbean root transcriptome (Figure 2). We noticed almost 1.5 times the number of DEGs at D7WS than D1WS (Figure 2A), demonstrating a significant transcriptome adjustment after D7WS. These results strongly suggest that the WS period intensely affects the adaptive root response. The reliability of RNA-seq results was confirmed by qRT-PCR (Figure 6).

This study applied comparative transcriptomic analysis for non-model species to identify the molecular mechanism responsible for waterlogging tolerance in mungbean. As expected, we found that DEGs commonly induced under D1WS and D7WS in both varieties involve hypoxia response (Figure 2B). However, numerous hypoxia-responsive genes were induced under D1KPS2/NSKPS2, D7KPS2/NSKPS2, and D1H150/NSH150 but not in D7H150/NSH150 (Figure 2B), demonstrating that H150 is better adapted to long-term WS than KPS2.

Adaptation to WS in plants involves the modification of diverse cellular processes. Our ORA results demonstrated the overview of how these processes are regulated by WS (Figure 3A,B). Starch degradation, glycolysis, and fermentative genes were induced in D1H150/NSH150 and D1KPS/NSKPS2. However, their induction was higher in D7KPS2/NSKPS2 than in D7H150/NSH150 (Figure 4A), indicating the change in root system architecture could improve internal root aeration.

As commonly known, plants’ response to WS relies on the induction of ethylene. In this study, ACC synthase, a key enzyme in ethylene biosynthesis, was more induced in D1H150/NSH150 than D1KPS2/NSKPS2 (Figure 3A,B and Figure 4B), implying that ethylene accumulation may occur faster and more robustly in D1H150/NSH150 than D1KPS2/NSKPS2. As root growth in *Arabidopsis* can be regulated by ethylene, which can locally activate the auxin signaling pathway by stimulating the auxin biosynthesis and transport machinery [19], a higher level of ethylene production in H150 could possibly enhance AR and LR production. To support this idea, the application of ethephon, a donor source of ethylene, enhanced the AR development of WS soybean (*Glycine max*). In mungbean, ethylene can promote AR formation of the cuttings [20].

In many plant species, root regeneration can be stimulated through wounding or cutting. Wounding can induce the formation of AR from non-root tissue. Wound response signaling pathways increase the JA level at the cut site, which results in AR formation [21]. In this study, the upregulation of ethylene-responsive transcription factors, *ABR1*, *ERF1*, *ERF112*, and *RAP2.6L*, was more pronounced in D1H150 than D1KPS2 (Figure 4B). In *Arabidopsis*, *ABR1* is involved in the wound and JA-induced auxin production by upregulating ANTHRANILATE SYNTHASE α1 (*ASA1*), a key enzyme in auxin biosynthesis, thereby promoting root growth and regeneration [22]. Similarly, *Arabidopsis ERF1*, an integrator of JA and ethylene signaling, can directly upregulate *ASA1* by binding to its promoter, leading to auxin accumulation and the inhibition of primary root elongation [23]. The *Arabidopsis RAP2.6L*, another wound, JA, and ethylene-induced transcription factor, has been shown to control various regenerative processes, including root stem cell maintenance and root growth [24,25]. Although the function of the *Arabidopsis ERF112* in root growth regulation has never been reported, it was placed in the ERFX subfamily similar to *ABR1* and *RAP2.6L* [26], implying they might have a redundant function.

A recent study in Arabidopsis demonstrated the increased levels of SQUAMOSA PROMOTER BINDING PROTEIN-LIKE 2 (SPL2), SPL10, and SPL11 could suppress root regeneration with age by inhibiting wound-induced auxin biosynthesis by repressing the expression of *ERF109* (a member of ERFX) and *ABR1* [22]. Interestingly, the *SPL* mRNAs are low-oxygen-induced microRNA156 (miR156) targets in many plant species [27,28,29]. In this study, *SPL2* (*Vradi02g11760*) expression was reduced upon WS in both H150 and KPS2 (Table S2), suggesting that the *miR156/SPL/ABR1* interaction might participate in the control of WS-triggered root plasticity in mungbean.

Previous studies demonstrated that low oxygen promotes the utilization of NO_3_^−^ and NO production, which can facilitate anaerobic survival [30,31]. Under low-oxygen, NO_3_^−^ could be used as NADH acceptors, allowing NAD^+^ to be reused in glycolysis. In this study, NR was more strongly expressed in D1H150/NSH150 and D7H150/NSH150 than D1KPS2/NSKPS2 and D7KPS2/NSKPS2 (Figure 5A). This could result in more robust NO production in H150 than in KPS2. Several pieces of evidence have shown that exogenous ethylene treatment increased endogenous NO levels via improving NR activity, which enhanced AR formation [32,33]. Taken together, higher NR expression in H150 than KPS2 may aid AR formation under WS.

Under WS, we observed an up-regulation of three PGBs (Figure 5A) in H150 and KPS2. However, the expression of all three PGBs was lower in D7H150/NSH150 than D7KPS2/NSKPS2, implying that at D7WS, the adaptation of the H150 root structure could provide more oxygen to the under-water roots. Concomitantly, the expression of low-oxygen responsive *ERFVIIs* and PCOs in both varieties was similar to the expression of PGBs. Most evolutionarily conserved core hypoxia-responsive genes [34,35] displayed a similar gene expression pattern. These results further demonstrated that H150 was better adapted to WS than KPS2.

**Figure 9:**
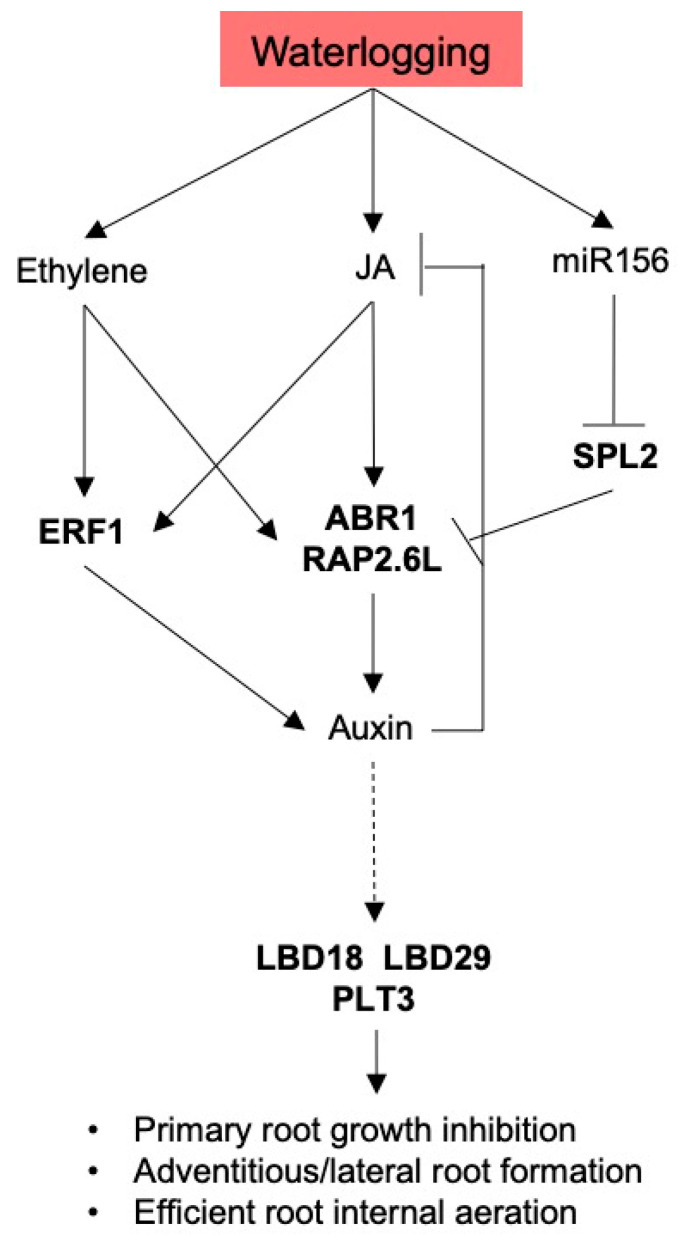
A proposed model of the molecular mechanism controlling WS-trigger root plasticity based on the transcriptome data.

As mentioned earlier, previous studies demonstrated that root regeneration could be regulated by wound and JA-induced ERF transcription factors. In this study, *ABR1* and *RAP2.6L* were strongly upregulated in D1H150/NSH150 but not in D1KPS2/NSKPS2 (Figure 8). Successively, auxin-regulated LR developmental genes, *CRF2, LBD18, LBD29, PLT3*, and *SMB,* were more highly expressed in H150 than KPS2 under D7H150/NSH150 than D7KPS2/NSKPS2 (Figure 8). These results further indicate that the wound and JA-induced ERFX transcription factors might serve as integrators of environmental signals that orchestrate root development in response to WS. We propose a model representing molecular mechanisms potentially involved in the WS-induced plasticity of root system architecture based on our transcriptome data (Figure 9).

## 4. Materials and Methods

### 4.1. Plant Material and Stress Treatment

*Vigna radiata* seeds, H150 (CN900198: landrace variety) and KPS2 (Kamphaeng Saen 2: commercial variety), were germinated in soil and grown outdoors between January and February 2020 at Kasetsart University, Bang Khen campus. Fifteen day-old, five-leaf-stage plants were used in the WS treatment. In brief, plant pots were placed in plastic containers filled with tap water. The level of water was set at 3 cm above the soil. WS began at mid-day. For the control, non-treated plants were placed in a container with no water. For each sample, the entire root tissue of six plants was harvested at the end of the treatment. It was immediately placed in liquid nitrogen, ground into a fine powder, and kept at −80 °C. For short-term and long-term WS, plants were subjected to WS for 1 day and 7 days, respectively.

### 4.2. Analysis of Root Phenotypic Plasticity

Adventitious roots that emerged from hypocotyls were counted at 0, 1, 3, 5, and 7 days after plants were subjected to WS. For the analysis of root morphology, underground roots were collected and photographed after 14 days of WS. Roots of NS plants grown side by side were used as controls.

### 4.3. RNA Extraction, Library Preparation, and Sequencing

Total RNA samples were extracted and subjected to DNase treatment and RNA cleanup using a GF-1 Total RNA Extraction Kit (Vivantis, Malaysia) according to the manufacturer’s protocol. Three replicates of total RNA samples were used for transcriptome analysis. The integrity of the RNA samples (RIN) was evaluated on an RNA 6000 Nano LapChiprun on Agilent2100 Bioanalyzer (Agilent Technologies, Germany). Samples with a RIN > 8.8 were used in RNA-seq library preparation. One μg of total RNAs was used to generate a sequencing library using an Illumina TruSeq Stranded mRNA LT Sample Prep Kit (Illumina, San Diego, CA, USA) following the manufacturer’s instructions. RNA-seq libraries were sequenced by an Illumina NovaSeq 6000 System according to the manufacturer’s instructions. Sequencing was carried out using a 2 × 100 bp paired-end (PE) configuration. Image analysis and base calling were conducted by the HiSeq Control Software (HCS) + RTA 2.7 (Illumina). Quality control filtering and 3′ end trimming were analyzed using the FASTX-toolkit (http://hannonlab.cshl.edu/fastx_toolkit/index.html, released date 5 January 2014) and Trimmomatic software (version 0.36) [36], respectively.

### 4.4. Differential Gene Expression Analysis

The FASTQ files were aligned to the mungbean reference genome [37] using HiSAT2 software (https://daehwankimlab.github.io/hisat2/, accessed on 6 March 2022, version 2.1.0) [38] with default settings. A binary format of sequence alignment files (BAM) was generated and used to create read count tables using the HTseq python library (https://htseq.readthedocs.io/, accessed on 6 March 2022, version 1.0) [39]. Differentially expressed genes were calculated using the edgeR program [40], with the GLM (generalized linear model) method using an FDR cutoff of <0.05. Significant DEGs were selected based on the FDR (<0.05) and at least having one comparison with a log2 fold change > 1 or <−1 (Table S2).

### 4.5. Ortholog Identification

Ortholog identification was performed using the predicted peptide sequences reported by Kang et al. [37] and *Arabidopsis thaliana* protein sequences (TAIR10) by OrthoVenn2 [41] using the default setting (e-value cutoff of 1e-2 for all protein similarity comparison and inflation value for the generation of orthologous clusters of 1.5). The orthologs can be found in Table S3.

### 4.6. Gene Ontology Enrichment Analysis

Gene ontology enrichment analysis was performed using the GOHyperGAll function [42] in the R environment. Mungbean-Arabidopsis ortholog Gene Ontology (GO) annotation was obtained from gene_association.tair.gz (https://www.arabidopsis.org/download_files/GO_and_PO_Annotations/Gene_Ontology_Annotations/gene_association.tair.gz, released date 1 January 2022). Significant GO terms were filtered by an adjusted *p*-value (FDR) of <0.05.

### 4.7. Overrepresentation Analysis

The mapping file was generated from the predicted peptide sequences reported by Kang et al. [37] using the Mercator pipeline. Over-representation analysis (ORA) was performed using the PAGEMAN program [43] with Fisher’s test.

### 4.8. Quantitative-Realtime PCR

Three replicates of total RNA samples were used. To eliminate contaminated genomic DNA, total RNAs were treated with DNase I (NEB, Ipswich, MA, USA). One microgram of total RNA was used to construct cDNA using MMuLv reverse transcriptase (Biotechrabbit, Berlin, Germany) in a final volume of 20 μL. The cDNA was diluted five times. Quantitative-realtime PCR (qPCR) was performed using QPCR Green Master Mix (Biotechrabbit) on a MasterCycler RealPlex4 (Eppendorf, Hamburg, Germany). For each sample, the PCR reaction was performed in triplicate. Each reaction contained 1 μL of diluted cDNA, 0.5 μM of each primer, and 10 μL of QPCR Green Master Mix in a final volume of 20 μL. The PCR cycle was 95 °C for 2 min, followed by 45 cycles of 95 °C for 15 s and 60 °C for 30 s. Amplification specificity was validated by melt-curve analysis at the end of each PCR experiment. Relative gene expression was calculated using the 2^−∆∆CT^ method. The genes and primers used are shown in Table S8.

### 4.9. Identification of Co-Regulated Genes by Clustering

Co-regulated genes were identified using fuzzy k-means clustering with Euclidean correlation for the distance measure, a membership exponent of 1.1, maximal number of iterations of 5000, and 30 clusters [44]. The mean SLR value for each cluster was determined for summary visualization (Table S6).

## 5. Conclusions

We aimed to elucidate the molecular mechanisms controlling WS tolerance using RNA-seq under short-term and long-term WS using two mungbean varieties with contrasting waterlogging tolerance and the ability to modify root system architecture. Our results demonstrated that the tolerant variety utilized an escape mechanism by modifying root architecture to improve low-oxygen conditions under WS. Under short-term WS, the expression of genes involved in metabolic adjustment towards the low-oxygen condition was similarly regulated in both varieties. However, ethylene, JA, and wound-responsive hub transcription factors that regulated root stem cells were strongly upregulated in the tolerant varieties. Under long-term WS, the expression of low-oxygen responsive genes was more upregulated in the sensitive variety. On the contrary, auxin-responsive root developmental genes were more highly expressed in the tolerant variety. We expect that the basic knowledge obtained from this study will help to improve our understanding of the mechanism linking the environmental signal to the developmental response and can be used for the molecular breeding of WS-tolerant mungbean and other crops in the future.

## Data Availability

The raw read files were deposited in the NCBI SRA database under the BioProject accession: PRJNA804574.

## Supplementary Materials

The following supporting information can be downloaded at: https://www.mdpi.com/article/10.3390/plants11070930/s1, Figure S1. CPM values of ACC synthase genes; Table S1. Read statistics; Table S2. Differentially expressed genes (DEGs) from seven comparisons: D1H150/NSH150; D1KPS2/NSKPS2; D7H150/NSH150; D7KPS2/NSKPS2; NSH150/NSKPS2; D1H150/D1KPS2; D7H150/D7KPS2.; Table S3. Orthologs between mungbean and Arabidopsis; Table S4. GO enrichment analysis derived from Venn’s diagram comparison.; Table S5. Overrepresentation analysis by PAGEMAN tool. Log_2_ Fold Change cutoffs: 1 and 2.; Table S6. The pattern of Fuzzy k-mean clusters; Table S7. GO enrichment analysis derived from Fuzzy k-mean clusters; Table S8. Primers used for quantitative real-time PCR.
