# Supplementary figures and images for: RNA-Seq Reveals Waterlogging-Triggered Root Plasticity in Mungbean Associated with Ethylene and Jasmonic Acid Signal Integrators for Root Regeneration
Source: Plants (Basel). 2022 Mar 30;11(7):930. doi: 10.3390/plants11070930 (PMC9002673; doi:10.3390/plants11070930)

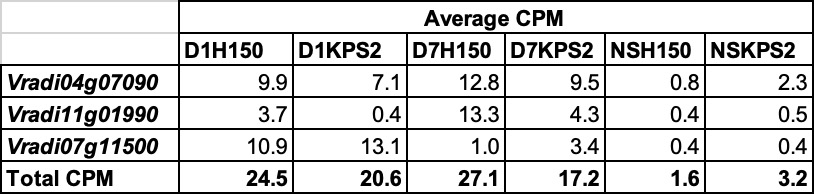

Supplement: Supplementary file 1 [file plants-11-00930-s001.zip › FigureS1.jpg]
